# Prevalence of inflammatory bowel disease among coeliac disease patients in a Hungarian coeliac centre

**DOI:** 10.1186/s12876-015-0370-7

**Published:** 2015-10-19

**Authors:** Dorottya Kocsis, Zsuzsanna Tóth, Ágnes A. Csontos, Pál Miheller, Péter Pák, László Herszényi, Miklós Tóth, Zsolt Tulassay, Márk Juhász

**Affiliations:** 1 2nd Department of Medicine, Semmelweis University, Szentkirályi st. 46., 1088 Budapest, Hungary; 2 Peterfy S. u. Hospital, Gastroenterology Unit, Péterfy S. st. 8-20., 1076 Budapest, Hungary; 3 Vaszary Kolos. Hospital, Gastroenterology Unit, Petőfi S. st. 26-28., 2500 Esztergom, Hungary

**Keywords:** Coeliac disease, Inflammatory bowel disease, Crohn’s disease, Ulcerative colitis

## Abstract

**Background:**

Celiac disease, Crohn disease and ulcerative colitis are inflammatory disorders of the gastrointestinal tract with some common genetic, immunological and environmental factors involved in their pathogenesis. Several research shown that patients with celiac disease have increased risk of developing inflammatory bowel disease when compared with that of the general population. The aim of this study is to determine the prevalence of inflammatory bowel disease in our celiac patient cohort over a 15-year-long study period.

**Methods:**

To diagnose celiac disease, serological tests were used, and duodenal biopsy samples were taken to determine the degree of mucosal injury. To set up the diagnosis of inflammatory bowel disease, clinical parameters, imaging techniques, colonoscopy histology were applied. DEXA for measuring bone mineral density was performed on every patient.

**Results:**

In our material, 8/245 (3,2 %) coeliac disease patients presented inflammatory bowel disease (four males, mean age 37, range 22–67), 6/8 Crohn’s disease, and 2/8 ulcerative colitis. In 7/8 patients the diagnosis of coeliac disease was made first and inflammatory bowel disease was identified during follow-up. The average time period during the set-up of the two diagnosis was 10,7 years. Coeliac disease serology was positive in all cases. The distribution of histology results according to Marsh classification: 1/8 M1, 2/8 M2, 3/8 M3a, 2/8 M3b. The distribution according to the Montreal classification: 4/6 Crohn’s disease patients are B1, 2/6 Crohn’s disease patients are B2, 2/2 ulcerative colitis patients are S2. Normal bone mineral density was detected in 2/8 case, osteopenia in 4/8 and osteoporosis in 2/8 patients.

**Conclusions:**

Within our cohort of patients with coeliac disease, inflammatory bowel disease was significantly more common (3,2 %) than in the general population.

## Background

According to the ESPGHAN definition, “Coeliac disease is an immune-mediated systemic disorder elicited by gluten and related prolamines in genetically susceptible individuals, characterised by the presence of a variable combination of gluten dependent clinical manifestations, coeliac disease specific antibodies, HLA-DQ2 and DQ8 haplotypes and enteropathy” [1]. However, coeliac disease (CeD) may occur at any age, with the potential involvement of any organs. The prevalence of CeD in Hungarian population ranges from 1 to 1,5 % [2]. In adulthood, “classic symptoms” including diarrhea, abdominal distension, malabsorption syndrome are frequently absent. Patients may exhibit minor gastrointestinal complaints, as well as numerous extraintestinal manifestations. For the diagnosis of CeD in dietary naive adult patients serological and histological examination are necessary. After diagnosis, the basis of all the present treatments for CeD is the life-long gluten-free diet.

Nowadays, the range of diseases that can be proven to occur more frequently in untreated CeD has expanded. Various autoimmune diseases show association with CeD, such as type-1 diabetes mellitus, autoimmune thyroid disorders, selective IgA-deficiency, autoimmune hepatitis Sjögren’s syndrome, juvenile chronic arthritis and the cutaneous form of celiac disease Dermatitis herpetiformis Duhring (DHD).

CeD and inflammatory bowel disease (IBD) are inflammatory disorders of the gastrointestinal tract with some common genetic, immunological and environmental factors involved in their pathogenesis. Several research showed that patients with CeD have a 5–10-fold risk of developing IBD when compared with that of the general population.

Fifteen years ago, an adult CeD centre was founded at Semmelweis University, 2nd Department of Internal Medicine, aiming to diagnose, care and follow-up CeD patients in a uniform manner. The number of treated adult patients has reached 300 by 2015, 50 % from Budapest and 50 % from the countryside. Thanks to the numerous presentations and publications on the activity of this adult CeD centre nationwide, this adult centre has gradually become a referral centre accepted and seeked by every gastroenterology outpatient settings in Hungary. Focusing on the closer region of our centre, the proportion of celiac patients ever recorded in this centre is estimated to be 30–40 % of all adult celiac patients diagnosed and living in the central region of Hungary, with a total number of inhabitants of approximately 2.5 million people. Concerning IBD, the same department is one of the 11 centres of Hungary, having a state permission to provide IBD patients with biological therapy. The aim of this study was to establish the prevalence of IBD in our coeliac centre over a 15-year-long study period.

## Methods

Our study is a retrospective analysis. The data collection, processing and evaluating of the results were performed according to the existing legislation and in accordance with the Declaration of Helsinki. According to Hungarian guidelines no special ethical approval was required for this retrospective study.

Results of totally 248 patients with CeD attending the coeliac centre of 2nd Department of Medicine at Semmelweis University from November of 1997 till November of 2013 were analysed. There were 193 female and 55 male patients (78 % vs. 22 %), with a mean age of 38 years, a median of 37 years and a range between 19 and 80 years. Of note, being a referral centre, in some cases, the diagnostic work-up of patients were launched in the primary care or at other settings. These patients were then referred to our centre for revision and confirmation of the suspected diagnosis. In order to diagnose CeD, tissue transglutaminase antibody testing (tTG; IgA and IgG normal range: 0–10 U/ml) and/or endomysium antibody tests (EMA) were used for serology. The IgG-tTG result was applied for cases of selective IgA-deficiency. Serological tests were carried out at Semmelweis University and the Coeliac Disease Center of Heim Pal Children’s Hospital. Duodenal biopsy samples were taken to determine the degree of mucosal injury described according to the modified Marsh classification. Histological evaluation was performed mostly at the 1st Department of Pathology and Experimental Cancer Research of the Semmelweis University.

To set up the diagnosis of IBD, clinical parameters, imaging techniques, colonoscopy, video capsule endoscopy and histology were applied. Dual energy X-ray absorptiometry for measuring bone mineral density (BMD) was performed on every patient. Statistical analysis was conducted using GraphPad InStat 3.0® (San Diego, CA) software.

## Results

In our material, 8/245 (3,2 %) CeD patients presented IBD. There were four males and four females, the mean age was 37 years, range 22–67 years. Crohn’s disease (CD) was present in 6/8 patients (three male, three female). Ulcerative colitis (UC) was present in 2/8 patients (one male, one female). In 7/8 patients the diagnosis of CeD was made first and IBD was identified during follow-up. The average time period during the setup of the two diagnosis was 10,7 years (median: 5 years, range 4–535 months). All the eight patients with both IBD and CeD had serologic evidence of CeD at time of initial diagnosis. Serological tests were performed with tTG in 2/8 cases, and EMA in 6/8 cases. The distribution of duodenal histology results according to Marsh-Oberhuber classification were 1/8 M1, 2/8 M2, 3/8 M3a, 2/8 M3b. The distribution according to the Montreal classification: 4/6 CD patients are B1 (nonstricturing, nonpenetrating) and 2/6 patients are B2 (stricturing), 2/2 UC patients are S2 (moderate UC). Biological therapy (infliximab) was administered to 2/8 patients. One patient had selective IgA deficiency, and one patient also suffered from DHD. Osteodensitometry was performed on every patient with CeD and IBD. Normal BMD was detected in 2/8 case (1/2 CD, 1/2 CU), osteopenia in 4/8 patients (2/4 CD, 2/4 CU), and osteoporosis in 2/8 patients (2/2 CD). The mean body mass index (BMI) was 21,53 kg/m^2^, range 17,28–23,94 kg/m^2^. Mean BMI for males was 22,25 and 20,74 kg/m^2^ for females, respectively (Table 1). All but one CeD-IBD patient had their CeD earlier diagnosed, than IBD; anemia was presented in totally four patients, diarrhea in two patients, loss of weight in two patients and one DHD in one patient, respectively. There was only 1/8 patient whose IBD was earlier diagnosed, in his case serological screening aimed at several other autoimmune disorder resulted tTG positivity.

**Table 1:** Clinical characteristics of patients with inflammatory bowel disease and celiac disease

Patients	Initial diagnosis	Second diagnosis	Time to second diagnosis	Coeliac serology	Marsh-classification	Montreal-classification	BMD (bone mineral density)
1	Crohn’s disease	Coeliac disease	~4 month	tTG +	M2	A2L3B1	osteopenia.
2	Coeliac disease	Ulcerative colitis	~9 month	tTG +	M3a	A2E3S2	osteopenia
3	Coeliac disease	Crohn’s disease	~8 years (96 month)	EMA +	M3b	A2L3B2	normal
4	Coeliac disease	Crohn's disease	~20 years (240 month)	EMA +	M1	A3L1B1	osteoporosis
5	Coeliac disease	Crohn's disease	~5 years 5 month (65 month)	EMA +	M3a	A2L2B2	osteopenia
6	Coeliac disease	Crohn's disease	~5 years (60 month)	EMA +	M2	A2L3B1	osteopenia
7	Coeliac disease	Crohn's disease	~44 years 7 month (535 month)	EMA +	M3b	A3L1B1	osteoporosis
8	Coeliac disease	Ulcerative colitis	~2 years 1 month (25 month)	EMA +	M3a	A2E2S2	normal

## Discussion

Several autoimmune diseases are associated with CeD. To date, no conclusive evidence is available that proves if the relationship between CeD and autoimmune diseases is mediated by gluten exposure, or if CeD and autoimmune diseases could occur together due to other causes, particularly the injury of the integrity of the intestinal barrier function and the common genetic background (Table 1) [3]. Genetic studies have identified four shared risk chromosomal loci: PTPN2, IL18RAP, TAGAP, and PUS10 in both diseases [4]. This considerable overlap between the associated genetic regions might indicate partial agreement of the pathogenesis both of these diseases. Patients with IBD and CeD have number of common symptoms like diarrhea, malabsorption, weight loss, long-standing history of iron deficiency anaemia, and loss of BMD and this could cause problems in the diagnostics [5–7].

The prevalence of CeD (1:100) multiply exceeds that of IBD (CD 0,1–16:10^4^, UC 0,5–25:10^4^) in general population [8]. Previous studies showed, that the prevalence of IBD is 5–10 times higher in CeD compared with that of the general population [9–12].

In our cohort of patients with CeD the prevalence of IBD was 3,2 %. Other studies carried out among adult coeliac patients also found a similar prevalence of IBD. *Yang* et al. reported the prevalence of IBD to be 2,20 % in a cohort of 455 CeD patients [13]. *Leeds at al.* presented an IBD prevalence of 3,30 % among 305 CeD patients, however the prevalence of CeD among IBD patients was comparable with that of the controls [14]. Furthermore*, Casella* et al. found lower risk of CeD in their cohort of IBD patients than in the general population [15]. Assumed cause of this difference is that the increased intestinal permeability in CeD may lead to increased antigen presentation and therefore generation of autoantibodies or increased bacterial translocation, which has been involved in IBD as a pathogenic mechanism [14, 16].

In our study, CD was more common than UC in a cohort of IBD patients. In contrast to our results, CeD were reported more frequently to be associated with UC than CD [10, 17–19]. Moreover, two further studies reported high prevalence of UC even among the first-degree relatives of patients with CeD [20, 21].

The average time period during the setup of the two diagnosis in our patients’ cohort was 10,7 years, this result correspond well with the results from a previous study [13].

In our setting, in patients with both CeD and IBD, initial CeD serological testing were performed in most of the cases with EMA, which are highly specific for CeD and are not found in IBD [22]. tTG can be false positive in some chronic disorders such as type 1 diabetes mellitus and chronic liver diseases. *Di Tola* et al. and *Farrace* et al. both detected increased tTG IgA antibodies levels in patients with CD and UC [23, 24]. These results suggest positive tTG autoantibodies may be a phenomen of autoimmunity and loss of specificity in CeD [25].

At the time of the diagnosis, the majority of our patients the duodenal biopsies presented low stage (M1, M2, M3a) according to Marsh-Oberhuber classification. Histological findings of Marsh 1 were consider not fully sufficient for the diagnosis of CeD, but the one of our patient, how presented M1 stage also had biopsy-proof Dermatitis herpetiformis Duhring, which is the skin manifestation of CeD. Casella and his colleagues observed similar distribution of histological results [15]. However, number of studies has found intraepithelial lymphocytosis and villous atrophy in CD patients without any evidence of CeD [26–28]. This histological status makes it difficult to differentiate CeD from CD.

In our patients’ cohort, the dominant behaviour of IBD was of the inflammatory type, which is in line with data in literature [14, 15, 29].

In our study, the mean body mass index value of 21,53 kg/m^2^ also falls within the normal range provided by the WHO. Unlike coeliac children, the vast majority of adult coeliacs are not malnourished, even overweight patients are not uncommon [30, 31]. However, malnutrition is a common feature of adult IBD [32]. In contrast to our results, low range of BMI in patients with both CeD and IBD has been reported [33–35].

IBD and CeD are associated with an increased risk of loss on BMD, caused by malabsorption and inflammation. Prevalence of osteoporosis were shown to be 7–35 % in CD patients, 18 % in UC patients, and 36–38 % in untreated in CeD patients, respectively [36–40]. Interestingly, the loss of BMD in our patients with two different disease potentially leading to malabsorption syndrome were not worse than for those suffering in only one of these disorders.

## Conclusions

In conclusion, within our cohort of patients with CeD, IBD was significantly more common (3,2 %) than in the general population. On this basis celiac disease patients can be considered as a new potential inflammatory bowel disease risk group. Diagnosis of CeD mostly preceded the diagnosis of IBD. The dominant behaviour of IBD was of the inflammatory type. In our study, CD was more common than UC among patients suffering from CeD. The mean BMI value of our sample is within normal range. The loss of BMD for our patients with CeD and CD were not worse than for those suffering in only one of these disorders.

## Abbreviations

BMD: Bone mineral density
BMI: Body mass index
CeD: Celiac disease
CD: Crohn disease
DEXA: Dual energy X-ray absorptiometry
EMA: Endomysium antibody
IBD: Inflammatory bowel disease
UC: Ulcerative colitis
tTG: Tissue transglutaminase antibody
